# The Results of Using a Transforaminal Lumbar Interbody Fusion Cage at the Upper Lumbar Level

**DOI:** 10.7759/cureus.15496

**Published:** 2021-06-07

**Authors:** Uzay Erdoğan

**Affiliations:** 1 Neurosurgery, University of Health Sciences, Bakırköy Prof. Dr. Mazhar Osman Training and Research Hospital for Neurology, Neurosurgery and Psychiatry, Istanbul, TUR

**Keywords:** disc herniation, interbody cage, lumbar-fusion, transforaminal, upper lumbar

## Abstract

Aim

The aim of this study is to apply surgical treatments to upper lumbar disc hernias in order to provide lumbar stability and lumbar lordosis using a transforaminal lumbar interbody fusion (TLIF) cage and to preserve the success rate of surgical results by protecting neural structures without excessive subject tension.

Material and methods

Between 2012 and 2017, 32 patients who had undergone an operation for upper lumbar disc herniation and who had received a transforaminal lumbar interbody fusion cage using a posterior technique were evaluated retrospectively.

Results

The radiological and clinical findings, surgical methods, and results of the patients were evaluated. In our study, 25 (78.1%) of the patients with upper lumbar disc hernias who were evaluated retrospectively were female and seven (21.9%) were male. Their average age was 55.43 years. The average follow-up was 21.75 months. The most common complaints were lower back pain, leg pain, and claudication. In the findings from neurological examinations, a positive result on the femoral stretching test occurred in 30 (93.7%) patients. In the degenerative spinal structure of patients at the L1-2 and L2-3 levels, a transforaminal lumbar interbody fusion was performed via a wide laminectomy with posterior stabilization due to a wide-bottomed disc hernia and stenosis. Only one of the patients with a neurological deficit still had a motor deficit after surgery.

Conclusion

While planning a surgery for upper lumbar disc hernias, the anatomical features of this region and the patients' radiological and neurological findings should be carefully evaluated. If TLIF is performed during upper lumbar region surgery, it may be preferable to perform it using a posterior technique.

## Introduction

In the course of their lives, between 60% and 80% of people complain of lower back pain [1]. Lumbar hernias are among the leading causes of lower back pain. Lumbar disc hernias are most common at the L4-5 and L5-S1 distances, and at least at the L1-2 level [2]. Upper lumbar disc hernias (ULDH) can cause different clinical complaints. They may cause back pain, leg pain, radiculopathy, urinary incontinence, paresis, saddle-type numbness, and autonomic dysfunction [2-5].

The anatomical structure of the upper lumbar region differs from that of the lower lumbar region. The presence of the conus medullaris in the upper lumbar region, the narrow canal structure, and the short lamina distances affect the surgical method and its success. Stabilization may be needed when facet joints need to be removed to ensure adequate decompression during surgical treatment.

The interbody fusion technique may be preferable for restoring the disc distance's height, reducing the rate of pseudoarthrosis, and providing lumbar sagittal alignment [6]. The transforaminal lumbar interbody fusion (TLIF) cage may be preferable among the interbody fusion techniques due to its ease of technical use, low rate of complications, and good fusion results [7].

In our study, we evaluated patients who applied to our clinic between 2012 and 2017, who had instability and wide-based disc herniation in the upper lumbar region, and who underwent posterior transpedicular stabilization using a posterior technique during TLIF. The surgical results were compared with those in the literature.

## Materials and methods

The patient population

The study was planned retrospectively and in accordance with the Helsinki Declaration and with the permission of our hospital's ethics committee. Ethics committee approval number 46082, dated November 2, 2015, was obtained from the Bakırköy Mental Health and Neurological Diseases Training and Research Hospital at the University of Health Sciences in Istanbul. Informed consent was obtained from all patients for their data to be used in the study.

Patients who underwent posterior stabilization and TLIF using a posterior approach at the upper lumbar level between 2012 and 2017 at a single center and who had a follow-up period of at least two years were retrospectively evaluated.

Neurological symptoms and complaints such as pain that did not resolve despite pain management methods (epidural injection, facet denervation, or physiotherapeutic rehabilitation) after at least three months included discopathy, instability, increased kyphosis, stenosis, and listhesis at the T12-L1, L1-2, and L2-3 levels and were correlated with MR findings. Patients who had stabilization surgery in these regions were included in the study.

We excluded patients who had previously undergone surgery in these regions, discectomy, or decompression without stabilization and who had a malignant disease, renal function loss, or a diagnosis of hyperproliferation in rheumatic disease. We also excluded those who had not undergone preoperative fusion using TLIF and those who did not give informed consent. Patients who were followed up for less than two years or who were no longer being followed up were also excluded from the study.

The ODI-VAS score for back pain

In the preoperative and postoperative evaluations of the patients, separate Oswestry Disability Index (ODI) and visual analog scale (VAS) scores for lower back and leg pain were obtained.

Method of preoperative radiological evaluation

The patients were evaluated by using the preoperative MR, CT, and X-ray findings. Using the MR result, the patient's illness was classified according to the findings of discopathy, stenosis, and listhesis at the T12-L1, L1-2, and L2-3 levels.

Perioperative management and surgical technique

All patients were operated on in the prone position under general anesthesia. Motor evoked potentials (MEP) and sensory evoked potentials (SEP) were followed by neuromonitoring in all patients. The area to be operated on was determined by fluoroscopy, and bilateral facets were revealed following the midline linear skin incision. Transpedicular screws were placed. A total laminectomy was performed because of the wide-based disc herniation and stenosis. At the distance level, both facets of both sides were taken in total. Bone structures were drilled into laterally with a motor up to the level of the lower and upper pedicles. Distraction was performed by combining the screws with the rod opposite the side where TLIF was to be performed. The root was lateralized and mobilized towards the upper pedicle. Thus, a large and sufficient area was obtained for a radical discectomy and the performance of TLIF. A radical discectomy was performed. The corpus surfaces were made ready for fusion by scraping the upper and lower vertebral endplates. Then, titanium or PEEK TLIF filled with autogenous bone graft was placed in the distance. The opposite rod was released. Compression was performed by placing bilateral rods to preserve the sagittal spine angulation. The release of the neural structures was confirmed by checking the bilateral foramina and the root course. The operation ended by conducting the final check using fluoroscopy.

Postoperative management

CT and radiographs were obtained for all patients postoperatively at three and six months and once every year following surgery. They were evaluated using VAS scores on the seventh day, third month, and first year post-operation. The functional results were evaluated by applying the ODI three months after surgery and in the first year of follow-up. The fusion was evaluated by an independent radiologist using 1-mm thin-slice CT scans at the final follow-up. Definite fusion was defined by trabecular bony bridges between adjacent vertebral bodies at the instrumented levels.

Complications

Intraoperative and postoperative short-term and long-term complications were evaluated.

Statistical analysis

The data obtained from the study were analyzed using the Statistical Package for the Social Sciences (SPSS) 22.0 program (IBM Corp., Armonk, NY). Descriptive information, that is, the frequency distributions and averages of the patients' clinical and demographic data, was obtained. A one-way repeated-measures analysis of variance (ANOVA) was conducted to examine the changes in the VAS and ODI scores over time, and dependent sample t-tests were applied post hoc for significant results. The significance level in the analysis was accepted as p = 0.05.

## Results

The patient population

The study included 25 (78.1%) female and 7 (21.9%) male patients. Their average age was 55.43 ± 7.94 years. The duration of the complaints of the patients was 14.03 ± 7.61 months and the follow-up period was 21.75 ± 9.96 months.

Among the patients, 19 (59.4%) patients with pre-operative lower back pain, 26 (81.25%) patients with leg pain, four (12.5%) patients with claudication, and 15 (46.9%) patients with neuropathic pain were classified as ill. In pre-operative neurological examinations, neurological deficits ranging from monoparesis to paraplegia were detected in eight (25%) patients, and a positive result on the femoral stretching test was obtained in 30 (93.7%) patients.

Preoperative radiological findings, their indications, and the plan

According to the MR findings, 29 (90.6%) of the patients were classified as having a discopathy, 20 (62.5%) had stenosis, and eight (25%) had listhesis. Of these patients, for 15 (46.9%), the location of their disorder was at L1-L2, for eight (24.9%), at T12-L2, for two (6.3%) at L2-L3, and for seven (21.9%), at L1-L3. The decision to stabilize was made based on the presence of a wide-based disc herniation, stenosis, and instability in the degenerative spinal structure of the patients.

Pre-postoperative management and the ODI-VAS score

The patients were evaluated using the ODI and VAS scores for postoperative back and leg pain. A one-way repeated-measures ANOVA was conducted to compare the changes in the patients' VAS (at the waist) scores over time. The VAS (at the waist) scores varied significantly over time [F (93, 3) = 36.112, p < .01] (Table 1).

**Table 1:** VAS (Waist) Point Average Comparison VAS: visual analog scale

			N	Average			SD		F (p)		
Pre-op			32	4,6250			3,81635		36,112 (,000)		
Post-op (7 days)			32	2,3750			,60907				
Post-op (3 months)			32	,9063			,85607				
Post-op (1 year)			32	,3437			,60158				
Note: F-Greenhouse-Geisser, df:93,3, SD: Standard Deviation											
VAS (Waist) Scores In-Group Comparisons											
	Level					Mean Square				F (p)	
VAS (Waist)	Pre-op vs. Post-op (7 days)					162,000				13,500(,001)	
	Post-op (7 gün) vs. Post-op (3 months)					69,031				107,166(,000)	
	Post-op (3 ay) vs. Post-op (1 year)					10,125				22,622(,000)	
Note: N:32, df:31,1											
Level Comparisons of VAS (Waist) Points											
(1) VAS (waist) Level		(2) VAS (waist) Level			Average Difference (1-2)			SE			p
Pre-op		Post-op (7 days)			2,250^*^			,612			,001
		Post-op (3 months)			3,719^*^			,599			,000
		Post-op (1 year)			4,281^*^			,653			,000
Post-op (7 days)		Pre-op			-2,250^*^			,612			,001
		Post-op (3 months)			1,469^*^			,142			,000
		Post-op (1 year)			2,031^*^			,138			,000
Post-op (3 months)		Pre-op			-3,719^*^			,599			,000
		Post-op (7 days)			-1,469^*^			,142			,000
		Post-op (1 year)			,563^*^			,118			,000
Post-op (1 year)		Pre-op			-4,281^*^			,653			,000
		Post-op (7 days)			-2,031^*^			,138			,000
		Post-op (3 months)			-,563^*^			,118			,000
Note: N: 32, SE: Standard Error, *: p <.01											

A one-way repeated-measures ANOVA was performed to compare the patients' VAS (at the leg) scores over time. The VAS (at the leg) scores varied significantly over time [F (93, 3) = 162.677, p < .01].

A one-way repeated-measures ANOVA was performed to compare the change in patients' ODI scores over time. The ODI scores varied significantly over time [F (62, 2) = 47.660, p < .01] (Table 2).

**Table 2:** ODI Point Average Comparison ODI: Oswestry Disability Index

			N	Average			SD			F (p)	
Pre-op			32	43,5000			30,53501			47,660 (,000)	
Post-op (3 months)			32	16,2500			4,89239				
Post-op (1 year)			32	3,9375			2,12417				
Note: F-Greenhouse-Geisser, df:62,2, SD: Standard Deviation											
ODI Scores In-Group Comparisons											
	Level					MeanSquare			F (p)		
ODI	Pre-op vs. Post-op (3 months)					23762,000			30,441 (,000)		
	Post-op (3 months)vs. Post-op (1 year)					4851,125			280,112 (,000)		
Note: N:32, df:31,1											
Level Comparisons of ODI Score											
(1) ODI Level		(2) ODI Level			Average Difference (1-2)			SE			p
Pre-op		Post-op (3 months)			27,250^*^			4,939			,000
		Post-op (1 year)			39,563^*^			5,164			,000
Post-op (3 months)		Pre-op			-27,250^*^			4,939			,000
		Post-op (1 year)			12,313^*^			,736			,000
Post-op (1 year)		Pre-op			-39,563^*^			5,164			,000
		Post-op (3 months)			-12,313^*^			,736			,000
Note: N:32, SE: Standart Error, *: p<.01											

In the findings from the postoperative neurological examinations, out of the eight (25%) patients who had neurological deficits, there was no improvement in one patient, and a partial increase in muscle strength was observed in one patient. Six patients recovered entirely as a result of a follow-up using physical therapy.

Complete fusion was observed in 30 (93.75%) patients in the CT one year postoperation while two (6.25%) patients had pseudofusion.

**Figure 1:**
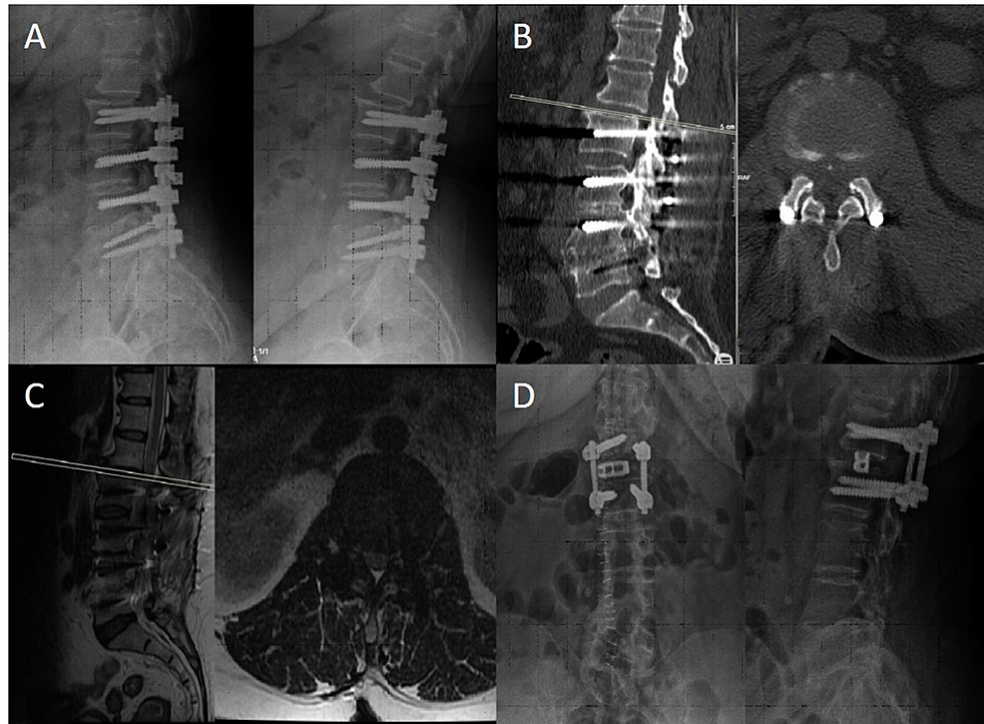
A) Preoperative Dynamic X-ray Images, B) Preoperative CT, C) Preoperative MRI, D) Postoperative Two-Way X-ray Images A-C: In the preoperative radiological images, it is seen that the adjacent segment, L1-L2 instability, and disc herniation developed in the patient who underwent L2-L3-L4-L5 posterior transpedicular stabilization. D: Since there was fusion in the lower segments, the transpeduncular screws in the lower segment were removed. Transpedicular stabilization of the L1-L2 level was performed with transforaminal interbody cage fusion. It shows the postoperative control X-ray.

**Figure 2:**
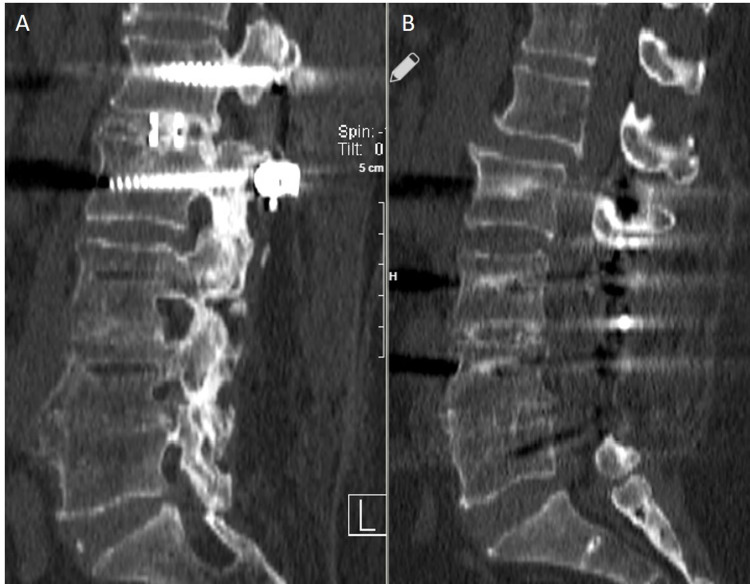
A: Postoperative 2nd Year CT, B: Preoperative CT A: Posterior transpeduncular stabilization and TLIF fusion are seen at the L1-L2 level. B: It is seen that the patient with an adjacent segment has instability at the L1-L2 level.

**Figure 3:**
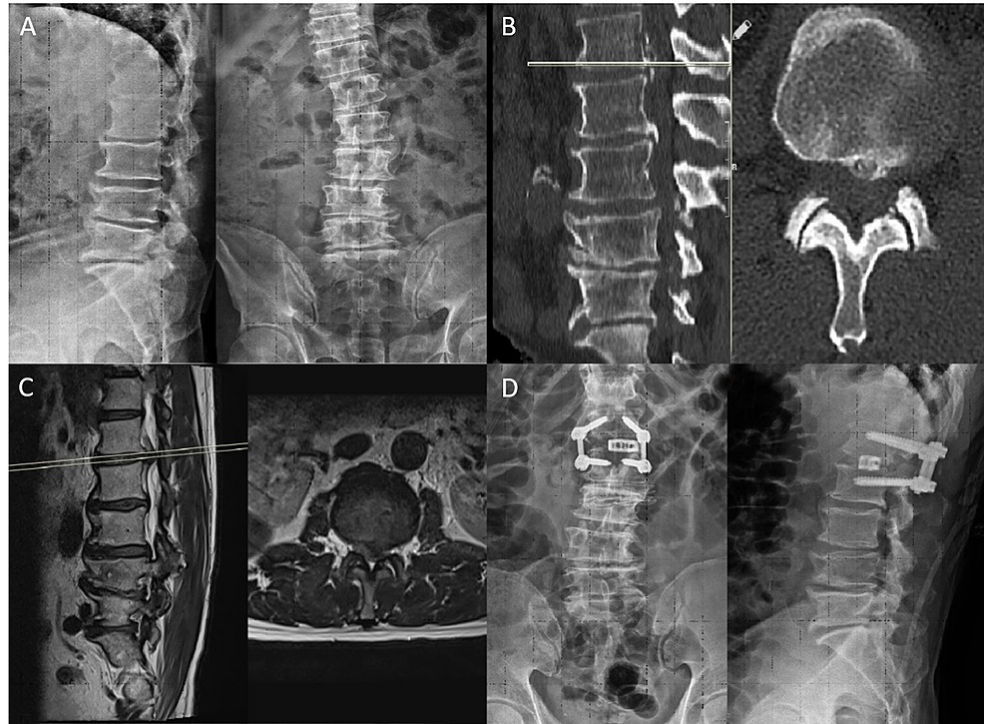
A: Preoperative Two-Way X-ray images, B: Preoperative CT, C: Preoperative MRI, D: Postoperative Two-Way X-ray Images

**Figure 4:**
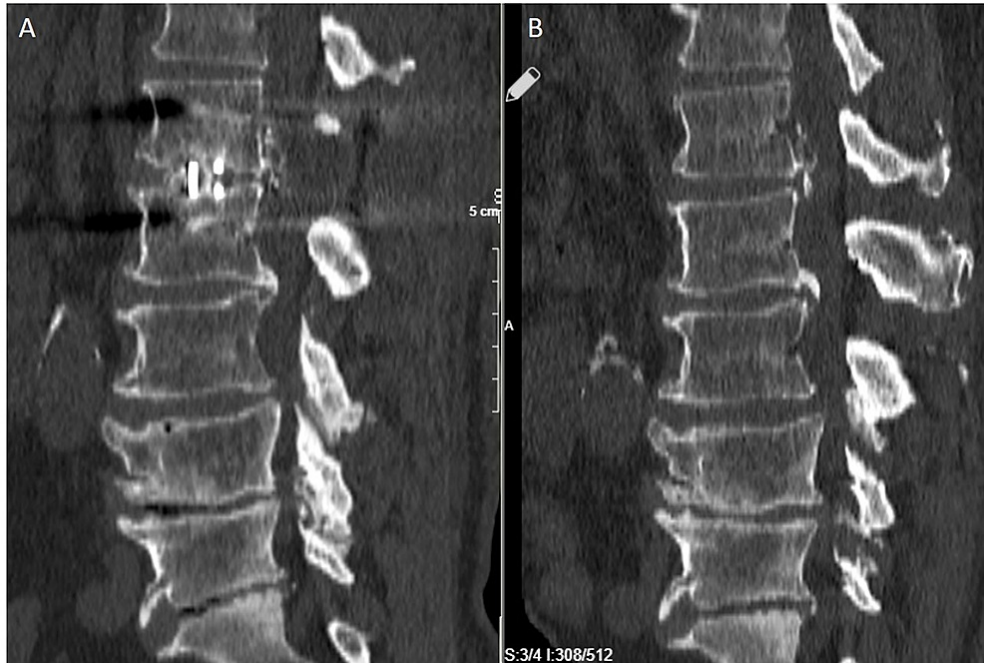
A: Postoperative 2nd Year CT, B: Preoperative CT

Radiological findings of two of the treated patients can be found in Figures 1-2 and Figures 3-4.

Complications and reoperations

In only one patient, the perioperative screw passed close to the medial pedicle. This was corrected in the same session to avoid any problems. No regression was noted using neuromonitoring. As an early complication, one (3.1%) patient who used an antiaggregant and an anticoagulant developed a postoperative hematoma and was operated on under emergency conditions to clear the hematoma. Serous discharge from the wound occurred in four (12.5%) patients one day post-operation. These discharges were brought under control by suturing under local anesthesia. In one (3.1%) patient, the wound site was revised after purulent discharge occurred postoperatively, and healing was achieved with antibiotic treatment. While pseudoarthrosis was observed in two patients as a long-term complication, one patient who had complaints was reoperated on, and their stabilization distance was extended. The screw broke in one patient due to trauma; therefore, the screws were removed. Adjacent segment disease did not develop in any of the patients during our follow-up.

## Discussion

The upper lumbar region comprises the distances defined as L1-2 and L2-3. The incidence of disc herniation in these segments constitutes approximately 1%-2% of all lumbar disc herniations [8]. While lower lumbar disc herniation is more common at 30-40 years of age and more common in men than in women, upper lumbar disc hernias are also seen at older ages [9]. Our study showed it to be more common in women (78.1%) than in men while the mean age was 55.43 ± 7.94 across both genders. Disc hernias located in the upper lumbar region may cause radiculopathy due to the narrow canal, cause disc hernias at the level of the conus medullaris in this region, and cause different complaints due to the compression of other nerve roots [4]. While urinary incontinence and paresis were most common in some studies [3], saddle-type numbness, back pain, and leg pain were the most common signs and symptoms in other studies [5]. In our study, the most common initial complaints were leg and back pain.

The low incidence of disc herniation in the upper lumbar region may cause a late diagnosis for this region [10]. Therefore, an anamnesis and the findings from a physical examination are essential for an early and accurate diagnosis. In the physical examinations of patients who had an upper lumbar disc herniation, the femoral stretching test was positive at the rate of 84-94%; this test is essential for diagnosing ULDH [11-12]. In our study, the femoral stretching test was positive in 93.7% of the patients' examinations, and this was consistent with the literature.

In radiological examinations, findings such as the disc's size and location, the degree of degeneration, and the spine alignment can be evaluated, and the appropriate surgical operation can be determined. The procedures for the treatments to be applied within this have been determined [13-15]. In patients with an upper lumbar disc herniation, if symptoms are bilateral and radiological findings support the complaints, a laminectomy can be performed bilaterally. When extensive decompression is needed, a medial facetectomy can also be performed [16]. In our study, patients who had stenosis, broad-based disc hernias, and increased kyphosis underwent a facetectomy with complete decompression. A TLIF was performed together with transpedicular stabilization to ensure instability and disc height.

Various surgical methods have been proposed for these pathologies. Endoscopic approaches have a lower incidence of operative morbidity compared to open surgery. Besides, less postoperative pain may be advantageous, with cosmetic benefits and rapid recovery [17]. However, the surgical results for upper-level lumbar disc hernias and their success rates are lower than for lower-level lumbar disc hernias. The reasons are that most of the disc hernias in this region have developed due to degeneration, the degeneration continues in the postoperative period, and the success rates may be lower due to conditions such as the narrowing of the disc space, the increase in kyphosis, and instability [18]. In our study, a posterior transpedicular stabilization and a posterior TLIF were performed in patients who had considerable kyphosis and an instability who underwent a wide laminectomy and a facetectomy. Our results were that 93.75% of the patients showed a long-term improvement in their complaints. Besides, statistically significant improvements were obtained in the patients' VAS and ODI scores from the postoperative period.

The muscles and ligaments around the vertebra are essential factors that ensure the stability of the spine. Weaknesses that occur in these structures may cause instability [19]. Spinal instability may also develop iatrogenically after surgery [20]. Spinal instability develops after a facetectomy, especially in the upper lumbar region since the pars intraarticular distance is narrow [21]. Despite these, lumbar fusion surgery is not routinely recommended for upper lumbar disc hernias, discectomies, or vertebral deformities [22]. Our results show that a posterior transpedicular stabilization and a posterior TLIF can be performed safely and effectively in patients who have considerable kyphosis and instability and who undergo an extensive laminectomy and a facetectomy.

Instability, kyphosis, and scoliosis due to degeneration in ligaments and facet joints can be seen in older people [23-24]. In some studies, a wedge deformation may occur in the spine due to endplate damage caused by degeneration, increasing kyphosis. Patients who have a kyphosis deformity are more likely to have a disc herniation in the upper lumbar region [25-26]. If there is an increase in the wedge deformation and kyphosis in the upper lumbar disc hernias, it should restore the spine alignment by adding an interbody cage to the stabilization process.

Various methods have been reported for interbody fusion: posterior lumbar interbody fusion (PLIF), transforaminal lumbar interbody fusion (TLIF or MI-TLIF), oblique lumbar interbody fusion or psoas anterior (OLIF/ATP), anterior lumbar interbody fusion (ALIF), and lateral lumbar interbody fusion (LLIF). The superiority of one approach and of its clinical results have not been demonstrated among these methods of fusion.

These procedures can also be performed using open or minimally invasive surgical (MIS) approaches. The anterior and anterolateral approaches are the most commonly used methods for interbody fusion [27]. ALIF is widely used to ensure segmental stability and intervertebral disc height [28]. It can be used alone or in conjunction with posterior fusion. When used alone, the spine is vulnerable to extension due to removing the anterior longitudinal ligament. Stabilization can be performed via the anterior and posterior routes to increase the fixation and fusion rate. The rate of complications increases in anterior and posterior fusions, and there are biomechanical changes in the spine. Besides, the duration of the operation is prolonged in anterior and posterior fusions, and increased surgical costs are their negative sides [29-30].

Due to the anatomical features of the upper lumbar region, it is believed that the application of TLIF posteriorly is complex and that the complications will be high. Some studies compared the anterior and posterior techniques of TLIF for different distances in the lower lumbar region, and no significant difference was found between the fusion results (7). In our patients who had considerable kyphosis and instability at the upper lumbar level, the TLIF posterior technique with a posterior transpedicular stabilization was performed. The two-year follow-up did not reveal any significant complications. Our fusion results were good. The application of TLIF using the posterior technique causes less bleeding and lower complication rates. Besides, there is the advantage of no significant retraction of the dura and conus medullaris.

Limitations

The patient population consisted of a heterogeneous group due to their pathologies. Following the same surgical procedure for quite different patients may limit the generalizability of our conclusions. The number of patients was limited and a more comprehensive assessment would result from examining more patients.

## Conclusions

For patients requiring upper lumbar segment decompression, interbody fusion can be added safely with this technique with no further morbidity. In this way, iatrogenic instability and the risk of pseudoarthrosis are eliminated. For upper segment listhesis patients, the posterior approach provides lower complications compared to the popular anterior-anterolateral technique. For both of these patient groups, the posterior approach can be a good alternative and should be included in preoperative approach planning options.
